# Gut *Lactococcus garvieae* promotes protective immunity to foodborne *Clostridium perfringens* infection

**DOI:** 10.1128/spectrum.04025-23

**Published:** 2024-08-27

**Authors:** Xue-Yin Wang, Fan-Hua Meng, Ming-Yue Zhang, Fen-Xin Li, Yu-Xin Lei, Zhao-Guo Ma, Jia-Qi Li, Ya-Nan Lou, Yue-Feng Chu, Ke Ma, Shui-Xing Yu

**Affiliations:** 1 State Key Laboratory of Reproductive Regulation and Breeding of Grassland Livestock, College of Life Sciences, Inner Mongolia University, Hohhot, China; 2 State Key Laboratory for Animal Disease Control and Prevention, College of Veterinary Medicine, Lanzhou University, Lanzhou Veterinary Research Institute, Chinese Academy agricultural Sciences, Lanzhou, China; 3 Inner Mongolia Engineering Technology Research Center of Germplasm Resources Conservation and Utilization, College of Life Sciences, Inner Mongolia University, Hohhot, China; Hubei University of Medicine, Shiyan City, Hubei, China

**Keywords:** probiotics, *Lactococcus garvieae*, intestinal mucosal barrier, *Clostridium perfringens*, enterocolitis

## Abstract

The gut microbiota, a pivotal component of the intestinal mucosal barrier, is critical for host resistance to enteric pathogen infection. Here, we report a novel function of the potentially probiotic *Lactococcus garvieae* strain LG1 (*L. garvieae* strain LG1) in maintaining intestinal mucosal barrier integrity and protecting against foodborne *Clostridium perfringens* (*C. perfringens*) infection. *L. garvieae* was isolated from the intestinal contents of Chinese Mongolian sheep (MS) and exhibited potential probiotic properties. In a *C. perfringens* enterocolitis model, *L. garvieae-*pretreated mice were less susceptible to *C. perfringens* infection compared with Phosphate buffered solution (PBS)*-*pretreated mice, which manifested as higher survival rates, lower pathogen loads, less weight loss, mild clinical symptoms and intestinal damage, and minor inflammation. Further mechanistic analysis showed that *L. garvieae* could ameliorate the disruption of intestinal permeability and maintain the integrity of the intestinal mucosal barrier by promoting the expression of tight junction proteins and mucoproteins. Moreover, *L. garvieae* was also able to facilitate antimicrobial peptide expression and ameliorate dysbiosis of the gut microbiota caused by *C. perfringens*. Together, these findings highlight the prospect of immunomodulatory potentially probiotic *L. garvieae* and might offer valuable strategies for prophylaxis and/or treatment of pathogenic *C. perfringens* mucosal infection.

**IMPORTANCE:**

*C. perfringens* necrotic enteritis leads to losses of about US $2 billion to the poultry industry worldwide every year. Worse, US Centers for Disease Control and Prevention (CDC) has estimated that *C. perfringens* causes nearly 1 million foodborne illnesses in the United States annually. Nowadays, the treatment recommendation is a combination of a broad-spectrum synergistic penicillin with clindamycin or a carbapenem, despite growing scientific concern over antibiotic resistance. The global understanding of the gut microbiome for *C. perfringens* infection may provide important insights into the intervention. *L. garvieae* originated from Mongolian sheep intestine, exhibited potentially probiotic properties, and was able to limit *C. perfringens* enterocolitis and pathogenic colonization. Importantly, we found that *L. garvieae* limits *C. perfringens* invasion via improving intestinal mucosal barrier function. Also, *L. garvieae* alleviates *C. perfringens*-induced gut microbiota dysbiosis. It allowed us to convince that utilization of probiotics to promote protective immunity against pathogens infection is of pivotal importance.

## INTRODUCTION

*Clostridium perfringens* (*C. perfringens*) is a Gram-positive, spore-forming, intestinal anaerobic bacterium (1, 2), and it has a high pathogenicity, causing food poisoning and non-foodborne diarrhea in mammals and poultry (3). Moreover, the US Centers for Disease Control and Prevention (CDC) have estimated that *C. perfringens* causes nearly 1 million foodborne illnesses in the United States every year (4). Thus, *C. perfringens* is considered to be one of the most common foodborne pathogens in the United States (2, 3, 5, 6). In addition, statistics show that subclinical and clinical necrotic enteritis caused by *C. perfringens* results in losses of about US $2 billion to the poultry industry worldwide every year (7–10). Currently, ceftiofur, penicillin, and erythromycin are still recommended for the treatment of *C. perfringens* infections, but there is a growing concern over antibiotic resistance globally (8, 11, 12). Therefore, there is an urgent need to develop alternative therapeutic strategies to combat *C. perfringens* infection.

Epithelial barrier integrity has a critical role in the maintenance of homeostasis in the body (13, 14), and barrier failure can cause inflammatory disorders and enhance the risk of chronic disease (15, 16). Similarly, the intestinal mucosal barrier, including biochemical and immunological components, acts as the first line of host defense against enteric pathogens (14, 17, 18). Beyond that, the commensal microflora in the intestinal mucosa has become more crucial in the causation and progression of intestinal diseases, which has led to great interest in using probiotics to regulate gut homeostasis to prevent or treat some diseases (19–22). In addition, probiotics derived from the commensal gut microbiota have been proposed as an important therapeutic strategy for inflammatory bowel disease treatment and have gradually become an alternative strategy to antibiotics.

*Lactococcus garvieae* (*L. garvieae*) is a facultative anaerobic, Gram-positive bacterium that is ubiquitous in nature. It is routinely regarded as a pathogenic bacterium that can cause a series of diseases in worldwide fish (including various farmed, wild marine, and freshwater species), such as body surface injury, liver anemia, and peritoneal hemorrhage (23, 24). However, several studies have confirmed that *L. garvieae* has potential probiotic properties, such as the ability to promote the growth of mice and broiler chickens and improve the intestinal flora of broiler chickens (25, 26). Furthermore, other studies show that *L. garvieae* can also produce the bioactive metabolite and prevent infection (27, 28). Nevertheless, the mechanisms by which *L. garvieae* protects the host against infection from pathogenic bacteria are still unknown.

In this study, we isolated an *L. garvieae* and evaluated its potential probiotic properties *in vitro*, further revealing the beneficial role of *L. garvieae* in protecting the host against *C. perfringens* infection. Mechanistically, the protective effect of *L. garvieae* against *C. perfringens* infection was dependent on its ability to potentially maintain intestinal mucosal barrier integrity and ameliorate the disruption of intestinal permeability. Meanwhile, we also found that *L. garvieae* could promote the expression of antimicrobial peptides to relieve the *C. perfringens*-elicited gut microbiota dysbiosis. Collectively, our results suggest that *L. garvieae* is essential for protecting the host against *C. perfringens* infection and may therefore contribute to providing scientific guidelines and laying an excellent foundation for the development and application of potentially probiotic *L. garvieae*.

## MATERIALS AND METHODS

### Mice and cells

Wild-type C57BL/6 J mice (6- to 8-week-old sex-matched mice) were purchased from the Model Animal Research Center of Inner Mongolia University (Hohhot, China). All experiments were conducted according to experimental practices and standards approved by the Animal Welfare and Research Ethics Committee of Inner Mongolia University [(2020) 022]. The human intestinal epithelial Caco-2 cell line was cultured in Dulbecco's modified eagle medium (DMEM) medium (Gibco, #12100046) containing 10% (vol/vol) fetal bovine serum (Gibco, #A31608-02).

### Determination of potential probiotic properties of *L. garvieae*

The suspension of *L. garvieae* was inoculated at 1% (vol/vol) into De Man, Rogosa and Sharpe (MRS) broth, and the OD_600 nm_ was measured at different temperatures (16°C, 23°C, 30°C, 37°C, 42°C, 49°C, or 56°C), different pH (2, 3, 4, 5, 6, 7, 8, or 9), or different concentrations of NaCl (0%, 1%, 3%, 5%, 7%, 9%, or 11%) under anaerobic conditions for 24 h. To further evaluate acid tolerance capacity, overnight cultures of *L. garvieae* were inoculated into either simulated gastric fluid (pH = 3 or 4) for 2 h and 4 h or simulated intestinal fluid containing bile salt (0%, 0.1%, 0.3%, 0.5%, or 1%) for 30 min, and the survivability was determined by counting on MRS agar under anaerobic conditions for 24 h. To evaluate adhere ability of *L. garvieae in vivo*, mice were orally administrated with 1 × 10^10^ Colony-forming units (CFUs) of ‌Fluorescein isothiocyanate (FITC)-tagged *L. garvieae* for 24 h. Scrape *the intestinal mucosa* to measure the fluorescence intensity using a Varioskan Flash plate reader (Thermo Scientific).

### Enterocolitis model

The *C. perfringens* enterocolitis model was established as previously described (29–31). To induce enterocolitis, wild-type C57BL/6 J mice (6- to 8-week-old sex-matched mice, *n* = 10 for each group) were orally exposed to a mixture of antibiotics (penicillin and streptomycin, 0.5 g/kg of body weight, gentamicin, 0.05 g/kg of body weight) for 2 days. Two days later, mice were inoculated orally daily with PBS or log-phase *L. garvieae* strain LG1 (a clinical isolate, NCBI GenBank accession number: OQ753881, 1 × 10^10^ CFUs/mouse) in a total volume of 200 µL prior to being orally challenged with 200 µL of a suspension containing 5 × 10^9^ CFUs of *C. perfringens* strain CP1 (a clinical isolate, NCBI GenBank accession number: MW440585). Aseptically excised tissues were homogenized. Serial dilutions of tissue homogenates were plated on Tryptose Sulfite Cycloserine (TSC) agar plates (Hopebio, #HB0253-9) containing 400 mg/L D-4-amino-3 isoxazolidinone antibiotics (Coolaber, #CA1662-1g) and bacterial loads (CFUs/g) were determined by colony counting after overnight anaerobic incubation. For the survival study, mice (6- to 8-week-old sex-matched mice, *n* = 10 for each group) were infected orally with 2 × 10^10^ CFUs of *C. perfringens* strain CP1.

### *In vivo* intestinal permeability

Permeability assay was detected using FITC-dextran as described previously (32). *L. garvieae* or PBS-pretreated mice were orally administrated with 5 × 10^9^ CFUs of *C. perfringens* for 24 h. Mice were gavaged with FITC-dextran (Sigma-Aldrich, #46944) at a dose of 600 mg/kg body weight for 4 h before harvest. The serum fluorescence intensity of the FITC-dextran was determined using a Varioskan Flash plate reader (Thermo Scientific) with an excitation wavelength of 490 nm and an emission wavelength of 530 nm.

### Tissue histology

The severity of enterocolitis was evaluated by body temperature measurements, body and cecum weight changes, and histologic analyses. Concisely, the body weight and temperature of mice were monitored daily. Tissue samples of the duodenum and cecum were fixed in a 4% buffered formalin solution (Macklin, #P804536), and paraffin sections were prepared. Subsequently, tissue sections were stained with hematoxylin and eosin (H&E, Solarbio, #SL7050-500) to examine morphologic changes. Duodenum and cecum histopathology scores were determined as previously reported (29, 30).

Caco-2 cells were incubated with PBS (2 h) alone, *L. garvieae* [Multiple of infection (MOI) = 50, 2 h] alone, PBS (30 min) before being stimulated with *C. perfringens* (MOI = 100, 1.5 h), or *L. garvieae* (MOI = 50, 30 min) before being challenged with *C. perfringens* (MOI = 100, 1.5 h). Cell death was analyzed by Annexin V-FITC/PI apoptosis detection kit (Beyotime, #C1062S) using a FACSAria flow cytometer (BD Biosciences) (33). In addition, cell viability was evaluated by the measurement of lactate dehydrogenase (LDH) leakage from damaged or destroyed cells (The LDH Cytotoxicity Assay kit, Beyotime Biotechnology, #C0017) as previously described (30).

### Cytokine and chemokine measurements

The tissues were mechanically homogenized in cold sterile PBS (4 mL/g tissue) containing a complete protease inhibitor cocktail (Sigma-Aldrich, #9036–19-5) and 1% Triton X-100 (Sigma-Aldrich, #93443). Tissue homogenates were then centrifuged at 12,000 rpm for 20 min. The supernatants were collected. Concentrations of Cytokines (IL-1β, IL-6, TNF-α) and chemokine (KC) were determined by Enzyme-linked immunosorbent assay (ELISA) (R&D Systems) according to the manufacturer’s instructions (30).

### Immunostaining

For immunohistochemistry, tissue sections were stained with F4/80 (1:100, BioLegend, #123119), Ly-6G/Ly-6c (1:100, BioLegend, #108419), Claudin 3 (1:200, Proteintech, #16456–1-AP), Occludin (1:200, Proteintech, #13409–1-AP), ZO-1 (1:200, Proteintech, #21773–1-AP), and Mucin 2 (1:200, Santa Cruz, #sc-515032) antibodies. Subsequently, specific staining was detected using the UltraSensitive S-P Kit (MXB, #KIT-9710) and DAB Detection Kit (Coolaber, #SK2020-3) according to the manufacturer’s directions (32). Additionally, mucin secretion and glycosylation patterns were stained and analyzed by using alcian blue/periodic acid-Schiff’s reagent (AB-PAS staining, Solarbio, #1285) following the manufacturer’s instructions as previously reported (30).

### Real-time PCR

Total RNA was extracted from the tissues using TRI-reagent (Sigma-Aldrich, #T9424). Quantitative real-time PCR assays were performed using SYBR Green (Roche, #04913914001) on an IQ5 Real-Time PCR Detection System (Bio-Rad). Gene expression levels were calculated using the 2^-ΔΔCt^ method. The primer sequences are as follows: GAPDH sense 5′-CACCCCAGCAAGGACACTGAGCAAG-3′, antisense 5′-GGGGGTCTGGGATGGAAATTGTT-GAG-3′, RegIIIβ sense 5′-TGTTACTCCATTCCCATCCACC-3′, antisense 5′-GTGCCTATGGCTCCTATTGCTA-3′, and RegIIIγ sense 5′-GTGCCTATGGCTCCTATTGCTA-3′, antisense 5′-ACCTCTGTTGGGTTCATAGCC-3′.

### Immunoblotting

Total protein was extracted as previously described (34). Western blot was performed using anti-Claudin 3 (1:1000, Proteintech, #16456–1-AP), anti-Occludin (1:1000, Proteintech, #13409–1-AP), anti-ZO-1 (1:1000, Proteintech, #21773–1-AP), and anti-GADPH (1:1000, Roteintech, #60004–1-Ig) antibodies.

### Gut microbiota assessment

Total microbial DNA was extracted from mouse feces using the Fast DNA Spin Kit (TransGenBiotech, #EE161-11). PCR amplification was carried out by using the V4 hypervariable regions of bacterial 16S rDNA. The purified amplicons were pooled in equimolar ratios and sequenced on the NovoMagic platform, and the sequenced data were analyzed using the QIIME 2 toolkit (35, 36).

### Statistical analysis

Data were statistically analyzed using GraphPad Software (La Jolla, CA, USA) and presented as mean ± SD. The differences between mean values were evaluated with Analysis of variance (ANOVA) (Dunnett’s or Tukey’s multiple comparison test) and Student’s *t*-test. Log-rank test is used for statistical analysis of animal mortality. *P-value was < 0.05 and **P-value was < 0.01 compared with the control group.

## RESULTS

### *L. garvieae* contributes to host protection against *C. perfringens* enterocolitis

**Fig 1:**
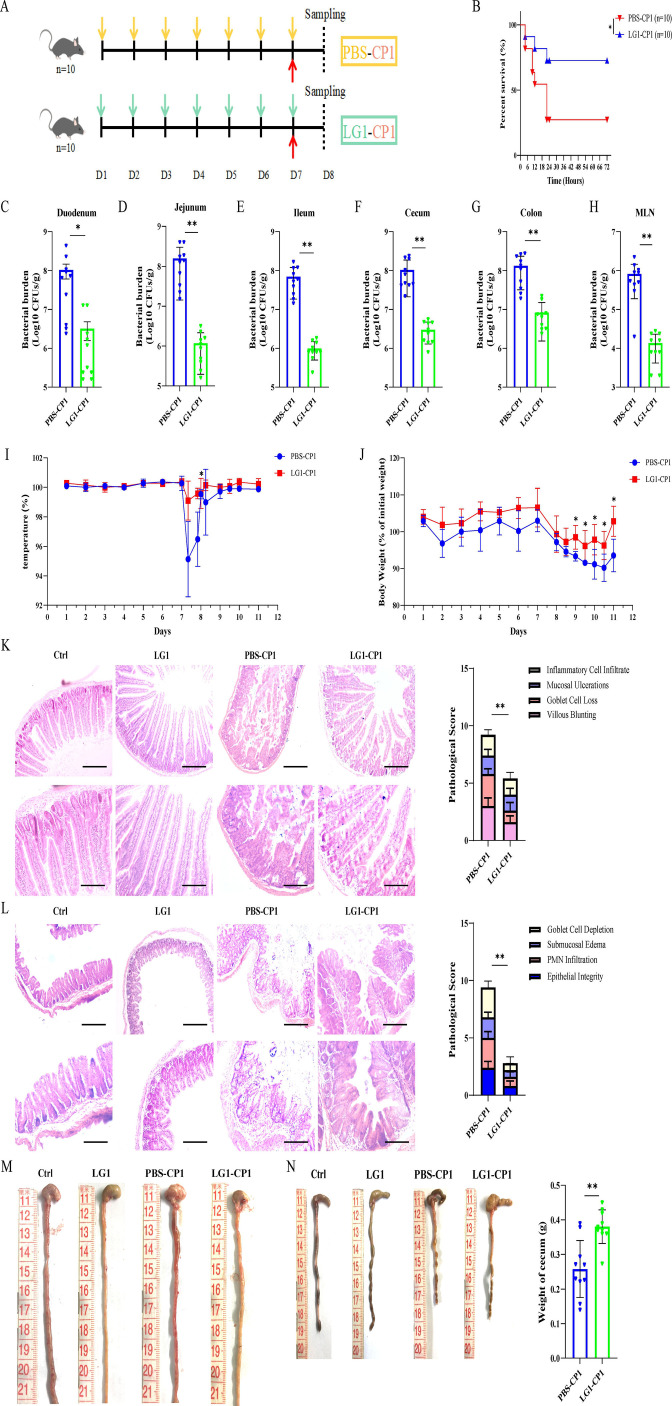
*L. garvieae* is efficient in protecting against *C. perfringens* enterocolitis. Six- to 8-week-old wild-type C57BL/6J mice (*n* = 10 for each group) were orally infected with log-phase *C. perfringens* strain CP1. (**A**) Sketch of experimental design. (**B**) Survival curve (2 × 10^10^ CFUs per mouse). (**C–H**) Bacterial burden in the duodenum, jejunum, ileum, cecum, colon, and MLN were quantitated (5 × 10^9^ CFUs per mouse, at 24 hpi). (**I**) Body temperature (5 × 10^9^ CFUs per mouse, mice were monitored over 12 days). (**J**) Body weight (5 × 10^9^ CFUs per mouse, mice were monitored over 12 days). (**K, L**) Representative pathologic photographs of H&E staining of the duodenum and cecum tissue of infected mice (5 × 10^9^ CFUs per mouse, at 24 hpi, upper panel, magnification ×100, lower panel, magnification ×200). (**M, N**) Representative gross appearance of the duodenum and cecum tissue, and weight of the cecum of infected mice (5 × 10^9^ CFUs per mouse, at 24 hpi). Graphs are means ± SD from data pooled from 10 (**A–N**) biological replicates. Data were considered significant when *P-value < 0.05 or **P-value < 0.01.

The bacterial isolate *L. garvieae* was obtained from the intestinal contents of Chinese Mongolian sheep (MS) and identified as *L. garvieae* (Fig. S1), and *in vitro* assessment of potentially probiotic properties revealed that *L. garvieae* had significant properties of potentially probiotic strains (Fig. S2 to S4). Furthermore, to explore the possible involvement of *L. garvieae* in the host response to *C. perfringens* infection, we initially constructed a *C. perfringens* mucosal infection model (Fig. 1A). Mice were challenged orally with PBS or *L. garvieae* (1 × 10^10^ CFUs per mouse) prior to oral administration of a lethal dose *C. perfringens* (2 × 10^10^ CFUs per mouse), and the mortality rates of mice were then monitored. Compared with PBS-pretreated mice, *L. garvieae-*pretreated mice demonstrated significantly increased survival (Fig. 1B). Moreover, PBS-treated mice and LG1-treated mice demonstrated an equivalent survival rate (Fig. S5). To evaluate if the decreased mortality in *L. garvieae-*pretreated mice was due to reduced pathogen loads, mice were administered orally with a lower infective dose of *C. perfringens* (5 × 10^9^ CFUs per mouse). Dramatically fewer *C. perfringens* were detected in the duodenum, jejunum, ileum, cecum, colon, and mesenteric lymph node (MLN) of *L. garvieae-*pretreated mice compared with PBS-pretreated mice at 24 h post-infection (hpi) (Fig. 1C through H). In accordance with the low level of pathogen burden, *L. garvieae-*pretreated mice exhibited milder clinical symptoms, exhibiting a small degree of body temperature changes and body weight loss (Fig. 1I and J). Simultaneously, this clinical assessment was validated by the histologic analysis of the duodenum and cecum. *L. garvieae-*pretreated mice displayed alleviative duodenal and cecal injuries and inflammation, indicated by mild destruction of epithelial integrity, submucosal edema, mucosal ulcerations, villous blunting, goblet cell loss, inflammatory cell infiltration, hyperemia, and hemorrhage (Fig. 1K through N). Collectively, these results suggest that potentially probiotic *L. garvieae* is required for host protection against *C. perfringens* mucosal infection.

### *L. garvieae* attenuates inflammatory responses upon *C. perfringens* mucosal infection

**Fig 2:**
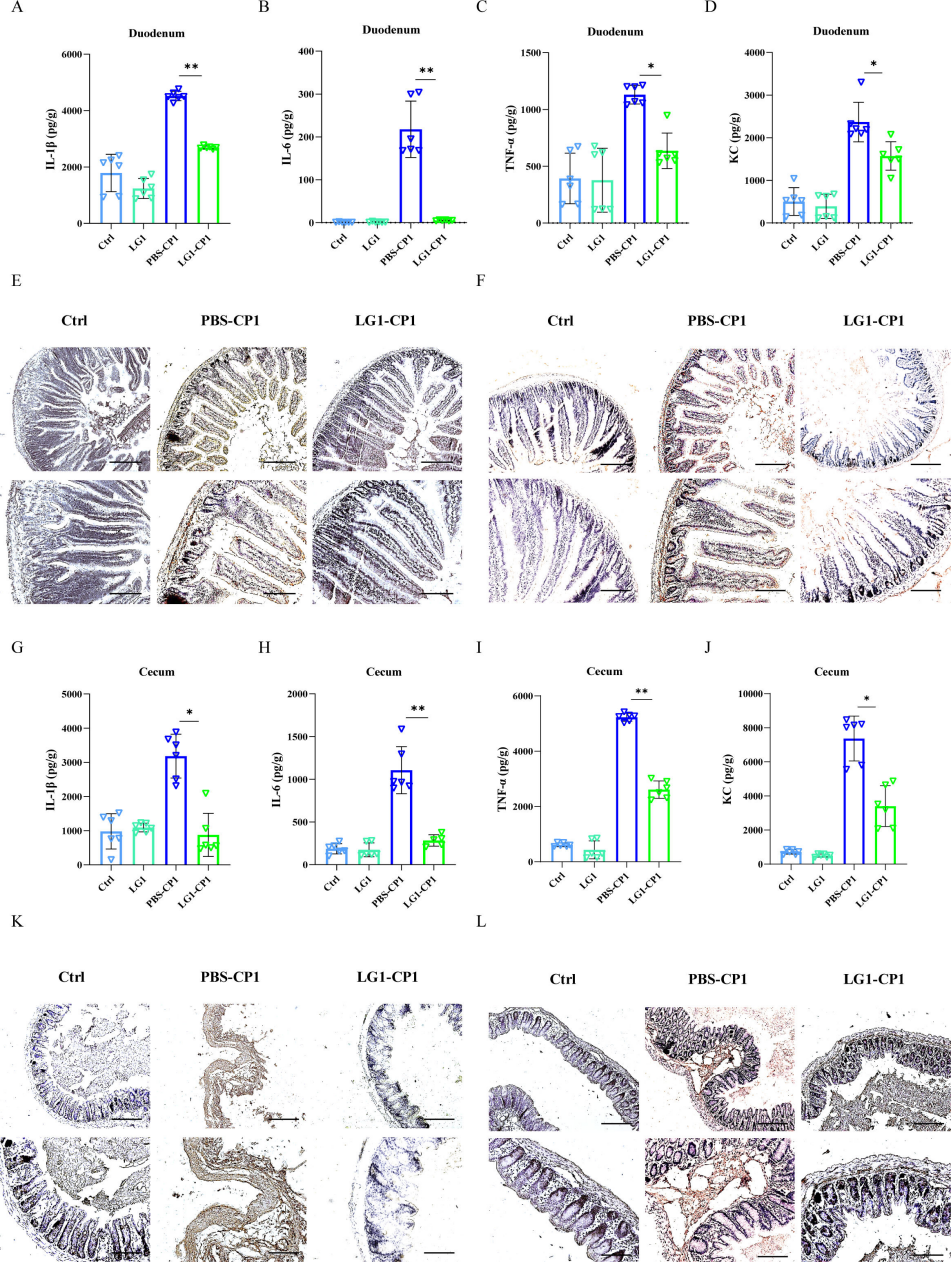
*L. garvieae* ameliorates inflammatory cell infiltration and pro-inflammatory mediator production during *C. perfringens* mucosal infection. Six- to 8-week-old wild-type C57BL/6J mice were infected orally with log-phase *C. perfringens* strain CP1 (5 × 10^9^ CFUs per mouse) for 24 h. (**A–D**) The homogenate supernatant of the duodenum tissue was analyzed for the presence of IL-1β, IL-6, TNF-α, and KC protein expression using ELISA. (**E**) Representative immunohistochemical staining F4/80 (a macrophagocyte marker) was performed in the duodenum sections (upper panel, magnification ×100, lower panel, magnification, ×200). (**F**) Representative immunohistochemical staining Gr-1 (a neutrophil marker) was performed in the duodenum sections (upper panel, magnification ×100, lower panel, magnification, ×200). (**G–J**) The homogenate supernatant of the cecum tissue was analyzed for the presence of IL-1β, IL-6, TNF-α, and KC protein expression using ELISA. (**K**) Representative immunohistochemical staining F4/80 (a macrophagocyte marker) was performed in the cecum sections (upper panel, magnification, ×100, lower panel, magnification, ×200). (**L**) Representative immunohistochemical staining Gr-1 (a neutrophil marker) was performed in the cecum sections (upper panel, magnification ×100, lower panel, magnification, ×200). Graphs are means ± SD from data pooled from ten (E, F, K, and L) or six (**A, B, C, D, G, H, I, and J**) biological replicates. Data were considered significant when *P-value < 0.05 or **P-value < 0.01.

To further determine the pathologic changes in the intestine, the secreted inflammatory cytokines and interstitial infiltration of inflammatory cells were examined. The results showed that at 24 hpi, amounts of inflammatory cytokines (IL-1β, IL-6, and TNF-α) and chemokines (KC) in the duodenum and cecum tissues of *L. garvieae-*pretreated mice were lower than in those of PBS-pretreated mice (Fig. 2A through D and G through J). Meanwhile, histologic and immunohistochemical analyses showed less neutrophil and macrophage accumulation in the duodenum and cecum tissue of *L. garvieae-*pretreated mice compared with PBS-pretreated mice (Fig. 2E, F, K, and L). Thus, these results indicate that potentially probiotic *L. garvieae* effectively suppresses intestinal inflammation associated with enhanced pathogen clearance during *C. perfringens*-triggered enterocolitis.

### *L. garvieae* restores the impairment of intestinal permeability

**Fig 3:**
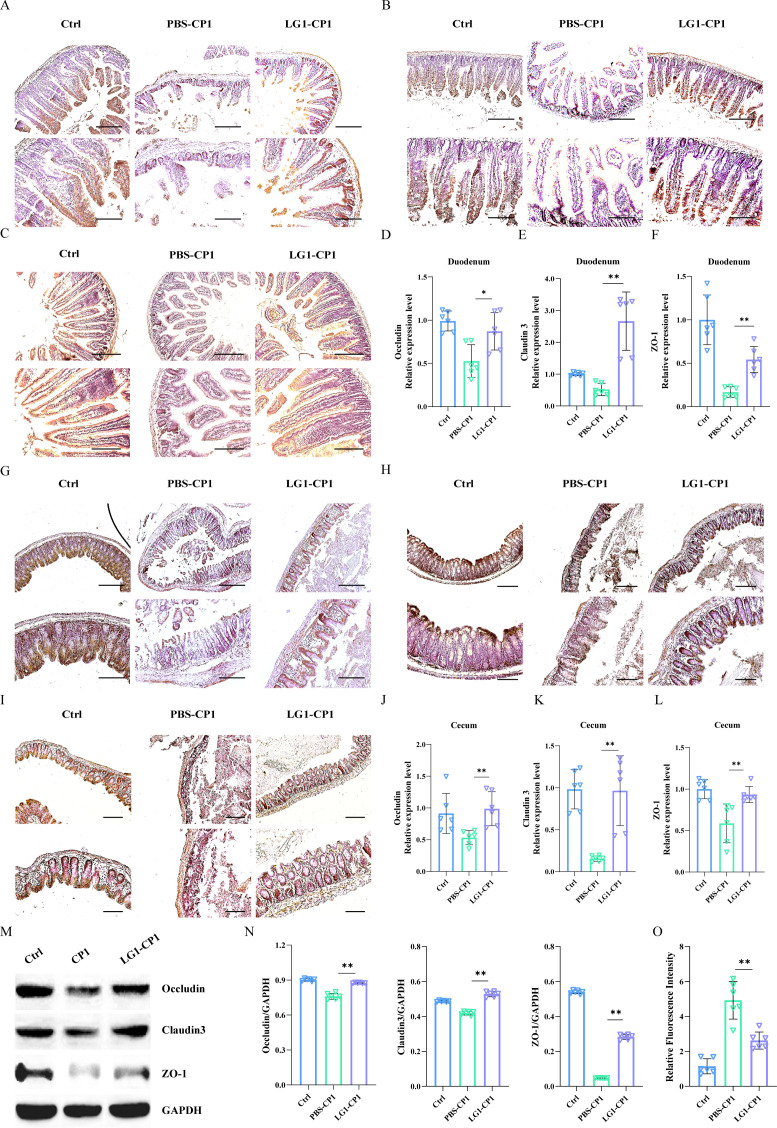
*L. garvieae* promotes the expression of tight junction proteins and maintains intestinal permeability. Six- to 8-week-old wild-type C57BL/6J mice were infected orally with log-phase *C. perfringens* strain CP1 (5 × 10^9^ CFUs per mouse) for 24 h. (**A–C**) Representative immunohistochemical staining of Occludin, Claudin 3, and ZO-1 was performed in the duodenum sections (upper panel, magnification ×100, lower panel, magnification, ×200). (**D–F**) The duodenum tissue mRNA was examined for the expression of Occludin, Claudin 3, and ZO-1 by qRT-PCR. The data were normalized to GAPDH expression and are shown as the fold increase in mRNA. (**G–I**) Representative immunohistochemical staining of Occludin, Claudin 3, and ZO-1 was performed in the cecum sections (upper panel, magnification ×100, lower panel, magnification, ×200). (**J–L**) The cecum tissue mRNA was examined for the expression of Occludin, Claudin 3, and ZO-1 by qRT-PCR. The data were normalized to GAPDH expression and are shown as the fold increase in mRNA. (**M, N**) The Caco-2 cell was stimulated with *L. garvieae* strain LG1 (MOI = 50, 30 min) prior to *C. perfringens* strain CP1 (MOI = 100, 90 min). The Caco-2 cell lysate was analyzed for the expression of Occludin, Claudin 3, and ZO-1 by western blotting. GAPDH was used as a loading control. (**O**) The paracellular intestinal permeability was determined using the fluorescent tracer FITC-dextran (600 mg/kg body weight, 4 h). Graphs are means ± SD from data pooled from 10 (A, B, C, G, H, and I) or six (D, E, F, J, K, L, M, N, and O) biological replicates. Data were considered significant when *P-value < 0.05 or **P-value < 0.01.

Next, we set out to seek the cause of the decreased *C. perfringens* burdens seen in *L. garvieae-*pretreated mice. The intestinal mucosal barrier is required for host protection against pathogenic intruders, and tight junctions are important constituents of the intestinal mucosal barrier. Thus, we initially investigated the effects of *L. garvieae* on tight junctions and paracellular intestinal permeability. Following the oral challenge of *C. perfringens*, there was a decrease in Occludin, Claudin 3, and Zonula occludens (ZO)-1 in the duodenum and cecum tissues of PBS-pretreated mice, whereas the groups pretreated with *L. garvieae* were significantly higher than those in the PBS-pretreated group and almost returned to control levels (Fig. 3A through L). Similarly, the expression of Occludin, Claudin 3, and ZO-1 had a higher level in *L. garvieae-*pretreated Caco-2 cells compared with cells pretreated with PBS (Fig. 3M and N). Subsequently, to further evaluate if *L. garvieae-*mediated protection was related to the preservation of intestinal barrier integrity, paracellular intestinal permeability was determined using the fluorescent tracer FITC-dextran. The fluorescence intensity of serum demonstrated that *L. garvieae-*pretreated mice displayed lower levels of FITC-dextran in blood than PBS-pretreated mice (Fig. 3O). Therefore, these results reveal a novel role for the potentially probiotic *L. garvieae* in maintaining intestinal barrier function during *C. perfringens* infection.

### *L. garvieae* promotes mucin and antimicrobial peptide synthesis

**Fig 4:**
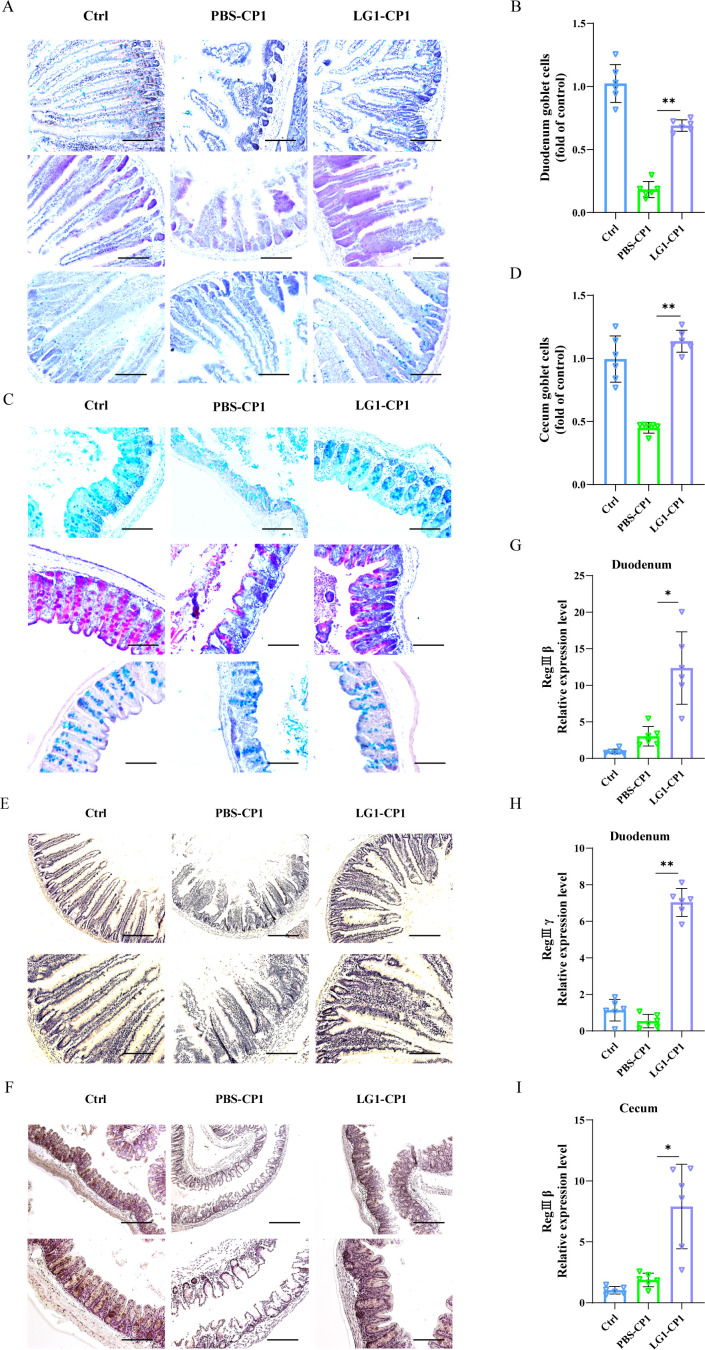
*L. garvieae* enhances the expression of mucoproteins and antimicrobial peptides. Six- to 8-week-old wild-type C57BL/6J mice were infected orally with log-phase *C. perfringens* strain CP1 (5×10^9^ CFUs per mouse) for 24 h. (**A, B**) Total mucin expression in goblet cells of duodenum tissue was examined via AB-PAS staining (magnification × 200). (**C, D**) Total mucin expression in goblet cells of cecum tissue was examined via AB-PAS staining (magnification × 200). (**E, F**) Representative immunohistochemical staining of mucin 2 was performed in the duodenum and cecum sections (upper panel, magnification ×100, lower panel, magnification, ×200). (**G, H**) The duodenum tissue mRNA was examined for the expression of RegIIIβ and RegIIIγ by qRT-PCR. The data were normalized to GAPDH expression and are shown as the fold increase in mRNA. (**I**) The cecum tissue mRNA was examined for the expression of RegIIIβ by qRT-PCR. The data were normalized to GAPDH expression and are shown as the fold increase in mRNA. Graphs are means ± SD from data pooled from 10 (**E, F**) or six (**A, B, C, D, G, H, and I**) biological replicates. Data were considered significant when *P-value < 0.05 or **P-value < 0.01.

As the mucus gel layer covering the epithelium is also crucial for intestinal mucosal barrier function, we suspected whether *L. garvieae* also promoted mucoproteins expression to inhibit *C. perfringens* infection. AB-PAS staining revealed that at 24 hpi, the expression of mucins was prominently larger in the duodenum and cecum tissues of *L. garvieae-*pretreated mice than that of PBS-pretreated mice, which almost returned to control levels (Fig. 4A through D). Compared with PBS-pretreated mice, mucin 2, a major component of the mucus layer, also trended to increase in *L. garvieae-*pretreated mice during *C. perfringens* infection (Fig. 4E and F). Furthermore, the expression levels of the antimicrobial peptides RegIIIβ and RegIIIγ were dramatically higher in the duodenum and cecum tissues of *L. garvieae-*pretreated mice compared with PBS-pretreated mice following *C. perfringens* infection (Fig. 4G through I). Hence, these findings indicate that potentially probiotic *L. garvieae* enhances mucoprotein and antimicrobial peptide expression and has a therapeutic effect on *C. perfringens* enterocolitis.

### *L. garvieae* alleviates *C. perfringens*-induced gut microbiota dysbiosis

**Fig 5:**
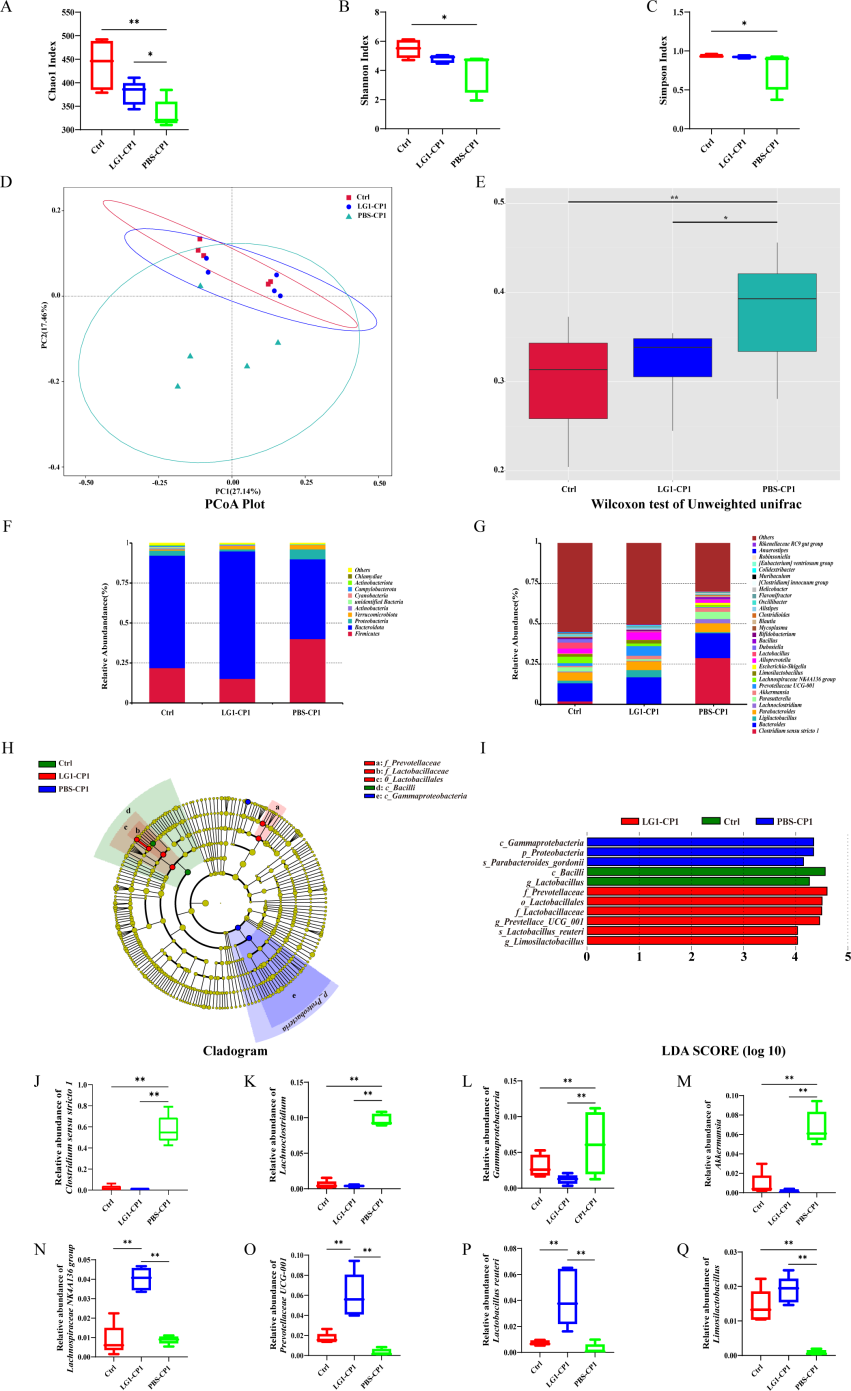
*L. garvieae* improves gut microbiota dysbiosis caused by *C. perfringens*. Effects of *L. garvieae* interventions on α-diversity and β-diversity of gut microbiota. (**A**) Chao 1 richness index of gut microbiota. (**B**) Shannon diversity index of gut microbiota. (**C**) Simpson diversity index of gut microbiota. (**D, E**) Unweighted Unifrac PCoA plot of gut microbial communities based on the OUT data. The microbial compositions were compared at different evolutionary levels. (**F**) The composition of the gut microbiota at the phylum level. (**G**) The composition of the gut microbiota at the genus level. (**H, I**) Cladogram of the linear discriminant analysis effect size (LEfSe) analysis of the gut microbiota. (**J–Q**) The relative abundance of gut microbial community representative members at the genus level. Graphs are means ± SD from data pooled from five (**A–Q**) biological replicates. Ctrl: Untreated Six- to eight-week-old wild-type C57BL/6J mice. LG1-CP1: Six- to 8-week-old wild-type C57BL/6J mice were orally exposed to a mixture of antibiotics (penicillin and streptomycin, 0.5g/kg of body weight, gentamicin, 0.05g/kg of body weight) for 2 days. Two days later, mice were inoculated orally daily with log-phase *L. garvieae* strain LG1 (1 × 10^10^ CFUs per mouse) in a total volume of 200 μL prior to orally challenged with 200 μL of a suspension containing 5 × 10^9^ CFUs of *C. perfringens* strain CP1. PBS-CP1: Six- to 8-week-old wild-type C57BL/6J mice were orally exposed to a mixture of antibiotics (penicillin and streptomycin, 0.5g/kg of body weight, gentamicin, 0.05 g/kg of body weight) for 2 days. Two days later, mice were inoculated orally daily with PBS in a total volume of 200 µL prior to orally challenged with 200 µL of a suspension containing 5 × 10^9^ CFUs of *C. perfringens* strain CP1. Data were considered significant when *P-value < 0.05 or **P-value < 0.01.

To further characterize the protective role of *L. garvieae* in mucosal defense against *C. perfringens*, we attempted to analyze gut microbiota to provide important insights into effective interventions with *L. garvieae.* Using the α-diversity analysis showed that the Chao1, Shannon, and Simpson indices of the gut microbiota were significantly decreased in the PBS*-*pretreated *C. perfringens*-infected mice compared with the PBS control mice, whereas these disturbance changes were reversed in the *L. garvieae-*pretreated *C. perfringens*-infected mice (Fig. 5A through C). According to the β-diversity analysis of the Unweight UniFrac Principal Co-ordinates Analysis (PCoA) algorithm, a clear separation was shown between the PBS control mice and PBS-pretreated *C. perfringens*-infected mice, whereas administration of *L. garvieae* strongly eliminated these differences (Fig. 5D and E). Next, fecal bacterial community profiles at different taxonomic levels were analyzed to give insights into specific differences in the gut microbiota resulting from the intervention of *L. garvieae.* At the phylum level, compared with the PBS control mice, the relative abundance of *Firmicutes*, *Proteobacteria*, and *Verrucomicrobiota* was dramatically increased in PBS-pretreated *C. perfringens*-infected mice, whereas the relative abundance of *Bacteroidota* was remarkedly decreased (Fig. 5F), indicating that challenge with *C. perfringens* resulted in the disturbance of gut microbiota in mice. However, *L. garvieae* treatment led to a reduced gut microbiota change at the phylum level (Fig. 5F). At the genus level, compared with the PBS control mice, the relative abundance of *Clostridium sensu stricto 1*, *Lachnoclostridium*, *Gammaprotebacteria,* and *Akkermansia* obviously increased, whereas the relative abundance of *Limosilactobacillus* dramatically decreased after *C. perfringens* challenge, and these trends were restored to similar levels after treatment of *L. garvieae* (Fig. 5G and J through Q). LEfSe analysis was used to identify the most differentially specific bacterial taxa that were predominant in PBS- and *L. garvieae-*pretreated *C. perfringens*-infected mice (Fig. 5H and I). Furthermore, results also demonstrated that the gut microbiota of the *L. garvieae-*pretreated *C. perfringens*-infected mice were enriched by *Lachnospiraceae NK4A136 group, Prevotellaceae UCG 001*, *Lactobacillus reuteri*, and *Limosilactobacillus*, which were more dominant in the gut microbiota of PBS control mice (Fig. 5N through Q). Conclusively, these results demonstrated that potentially probiotic *L. garvieae* could restore the alterations in intestinal microbial community structures to alleviate gut microbiota dysbiosis caused by *C. perfringens*.

## DISCUSSION

*C. perfringens,* an important foodborne pathogen, is a serious threat to public health and the animal industry (1, 11). Although *C. perfringens* is sensitive to penicillin, chloramphenicol, tetracycline, and ampicillin, there is a growing concern over antibiotic resistance globally (8, 37). Thus, the development of new preventive and therapeutic strategies to combat *C. perfringens* infection has become very urgent (38). The intestinal mucosal barrier, consisting of mechanical, chemical, immunological, and microbial barriers, can restrict mucosal colonization by pathogens and also resist penetration by pathogens, whereas probiotics are beneficial in reducing intestinal mucosal damage and permeability. Therefore, research on the potentially probiotic *L. garvieae* is critical for the design of potential therapeutic interventions against *C. perfringens* infection.

*L. garvieae* was routinely considered an emerging zoonotic pathogen. As the causative agent*, L. garvieae* can induce hemorrhagic septicemia in fish and cause severe fiscal loss in the aquaculture industry (23, 24). Meanwhile, the number of reports of *L. garvieae* isolates associated with fish disease has increased worldwide (27, 39). Most people were infected by *L. garvieae* after possibly ingesting raw seafood with infection. However, there were some different sounds. *L. garvieae* has been proven to have probiotic properties such as high tolerance to gastrointestinal juices, native gut commensal, optimizing the structure of gut flora, and promoting growth performance, which is expected to make it a novel probiotic candidate (25, 27, 40). Previous research has shown that *L. garvieae* FUA009 possesses the capability to produce the bioactive metabolite urolithin A from ellagic acid (27). Meanwhile, *L. garvieae* B301 relieves the incidence of diarrhea among broilers (25). In line with these, *L. garvieae* was isolated from Chinese Mongolian sheep (MS), and *in vitro* assessment of potentially probiotic properties revealed that *L. garvieae* had significant properties of potentially probiotic strains and may play an important role in host defense against *C. perfringens* mucosal infection. Therefore, we defined a novel role for *L. garvieae* in the host’s defense against pathogen infection.

Subsequently, we explored the mechanisms by which *L. garvieae* participated in host resistance to *C. perfringens* mucosal infection. Evidence has demonstrated an important role for tight junction proteins in gut disease, and tight junction proteins are also considered a hallmark in disease biology (41–44). In addition, antimicrobial peptides produced by intestinal epithelial cells and Paneth cells are a crucial part of the intestinal mucosal innate immune barrier (45, 46). For example, the antimicrobial peptides RegIIIβ and RegIIIγ possess bactericidal activity (47, 48). Obviously, there is growing evidence that indicates probiotics are involved in maintaining intestinal barrier function. *Lactobacillus rhamnosus* GG combined with inosine alleviates the damage to intestinal structure and intestinal tract permeability and increases tight junction protein expression in the alcohol-induced liver disease (ALD) mouse mode (49, 50). *Bifidobacterium* promotes intestinal barrier function and improves intestinal tight junction protein integrity in a rat neonatal necrotizing enterocolitis (NEC) model (51). Moreover, gut microbiota-derived propionate regulates the expression of RegIII mucosal lectins and ameliorates experimental colitis in mice (52). Exhilaratingly, our results also revealed that *L. garvieae* pretreatment could significantly improve *C. perfringens*-induced intestinal barrier injury and increase the expression levels of tight junction proteins and antimicrobial peptides RegIIIβ and RegIIIγ. Hence, these findings indicate that a likely mechanism of the resistance of potentially probiotic *L. garvieae* to *C. perfringens*-induced enterocolitis is the ability to maintain gut barrier integrity and antimicrobial peptide expression.

Substantial evidence suggests that dysregulation of the gut microbiota can disrupt the intestinal barrier and imbalance intestinal immunity, thereby contributing to the infection of pathogens. Probiotic supplementation can alter gut microflora and maintain intestinal homeostasis and the mucosal barrier (35). Artificial enzymes armed *Bifidobacterium longum* probiotics for alleviating intestinal inflammation and restoring the gut microbiota in inflammatory bowel disease (53). *Bifidobacterium breve* CCFM1078 relieves collagen-induced arthritis through modulation of the gut microbiota and repairing the intestinal barrier injury (54). *Lactobacillus acidophilus* ameliorates obesity via modulating gut microbiota dysbiosis and intestinal permeability, including significantly increasing the relative abundance of *Faecalibaculum*, *Bifidobacterium,* and *Lactobacillus*, and reducing the relative abundance of *Lachnospiraceae NK4A136 group* (55). Simultaneously, supplementation of broiler diets with *L. garvieae* B301 resulted in an increase in the number of caecum lactic acid bacteria and *Bifidobacterium spp.* and a decrease in the number of caecum coliforms (25). Correspondingly, our results demonstrate that *L. garvieae* partially reversed *C. perfringens*-induced changes in gut microbiota composition according to α-diversity and β-diversity analyses. Notably, at the genus level, *L. garvieae*-pretreated mice evidently increased the relative abundance of *Lachnospiraceae NK4A136 group*, *Prevotellaceae UCG-001*, *Lactobacillus reuteri*, and *Limosilactobacillus* while pronouncedly reducing the relative abundance of *Gammaprotebacteria*. Actually, it has been reported that the increase in the relative abundance of *Gammaprotebacteria* would lead to gut microbiota disorder and intestinal mucosal barrier damage (56). Probiotic *Lactobacillus* was reported to inhibit pathogens invading, stimulate mucin secretion, and control inflammation (57). *Prevotellaceae* can secrete lactic acid, improve the colonization of *Lactobacillus* in the intestine, and maintain the integrity of the intestinal barrier (58). Thus, *L. garvieae* could alleviate the disruption of intestinal barrier function through modulation of the gut microbiota. Importantly, previous studies point to the protective effects of *Saccharomyces boulardii* protease on *Clostridium difficile* toxins A and B in human colonic mucosa (59). Meanwhile, *Bacillus clausii* and *Lactobacillus reuteri* secrete compounds including serine protease, bacteriocin, and nonprotein antimicrobial compounds directly inhibiting *Clostridium difficile* infection (60). Consistent with these findings, our results show that the protective role of *Lactococcus garvieae* strain LG1 in maintaining intestinal barrier integrity to limit early bacterial colonization against *C. perfringens* enterocolitis. Based on our results and other reports, probiotics have been proposed as an important therapeutic strategy for the treatment of *Clostridium* infections and have gradually become an alternative strategy to antibiotics.

In summary, the present study demonstrated that *L. garvieae* protects against *C. perfringens* mucosal infection by promoting intestinal mucosal barrier function in mice. The results further confirmed the beneficial role of *L. garvieae* in host defense against *C. perfringens* infection. Moreover, *L. garvieae* might be a strong candidate for future probiotic development, and it also provides a scientific basis for the development of microecological agents for the prevention and treatment of *C. perfringens* in mammals.

### Conclusions

Our study provides an unexpected discovery of gut potentially probiotic *L. garvieae* in the context of foodborne *C. perfringens* infection, which contributes to the maintenance of intestinal barrier integrity and host defense. We provided a certain clue that potentially probiotic *L. garvieae* facilitates pathogen control by promoting intestinal mucosal barrier function. Also, potentially probiotic *L. garvieae* restores the alterations in intestinal microbial community structures to alleviate gut microbiota dysbiosis caused by *C. perfringens*. Additional studies are needed to a deeper understanding of the underlying mechanisms of potentially probiotic *L. garvieae* regulating intestinal mucosal barrier function, which is critical for the design of potential therapeutic interventions.

## Data Availability

The 16S rRNA sequence of *L. garvieae* strain LG1 and *C. perfringens* strain CP1 are available in GenBank with the accession OQ753881 and MW440585.

## SUPPLEMENTAL MATERIAL

Fig. S1 spectrum.04025-23-s0001.pdf Neighbor-joining Phylogenetic tree showing the relative position of the isolated LG1 based on the 16S rDNA sequences.

Fig. S2 spectrum.04025-23-s0002.pdf Determination of physiological properties of *L. garvieae* LG1.

Fig. S3 spectrum.04025-23-s0003.pdf Evaluation of the ability of *L. garvieae* LG1 to adhere to the intestinal mucosal surface.

Fig. S4 spectrum.04025-23-s0004.pdf The effect of *L. garvieae* LG1 on the viability of human intestinal epithelial Caco-2 cells.

Fig. S5 spectrum.04025-23-s0005.pdf The survival curve of the experimental scheme.

ASM does not own the copyrights to Supplemental Material that may be linked to, or accessed through, an article. The authors have granted ASM a non-exclusive, world-wide license to publish the Supplemental Material files. Please contact the corresponding author directly for reuse.
