# Supplementary material for: Gut Lactococcus garvieae promotes protective immunity to foodborne Clostridium perfringens infection
Source: Microbiol Spectr. 2024 Aug 27;12(10):e04025-23. doi: 10.1128/spectrum.04025-23 (PMC11448249; doi:10.1128/spectrum.04025-23)
Supplement: Fig. S1 — Neighbor-joining Phylogenetic tree showing the relative position of the isolated LG1 based on the 16S rDNA sequences. [file spectrum.04025-23-s0001.pdf]

Figure S1

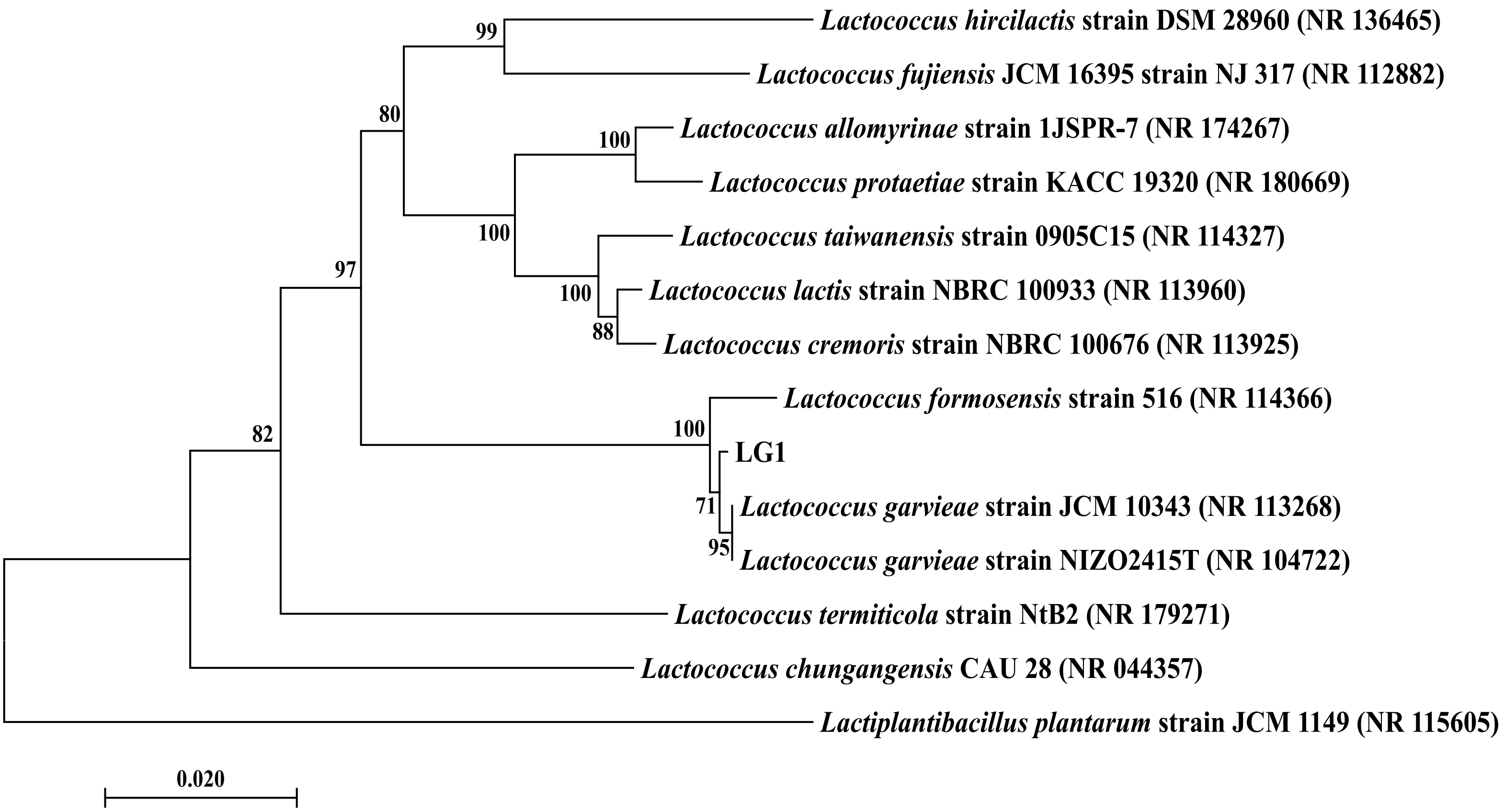

Figure S1. Neighbor-joining phylogenetic tree showing the relative position of the isolated *L. garvieae* LG1 based on the 16S rRNA sequences. Bootstrap values (1000 replicates) are indicated at branch nodes and the respective GenBank accession numbers for 16S rRNA genes are indicated in parenthesis. The bar represents 0.02 substitutions per nucleotide position. Lactiplantibacillus plantarum strain JCM 1149 is presented as the outgroup.
