# Supplementary material for: Gut Lactococcus garvieae promotes protective immunity to foodborne Clostridium perfringens infection
Source: Microbiol Spectr. 2024 Aug 27;12(10):e04025-23. doi: 10.1128/spectrum.04025-23 (PMC11448249; doi:10.1128/spectrum.04025-23)
Supplement: Fig. S2 — Determination of physiological properties of L. garvieae LG1. [file spectrum.04025-23-s0002.pdf]

Figure S2

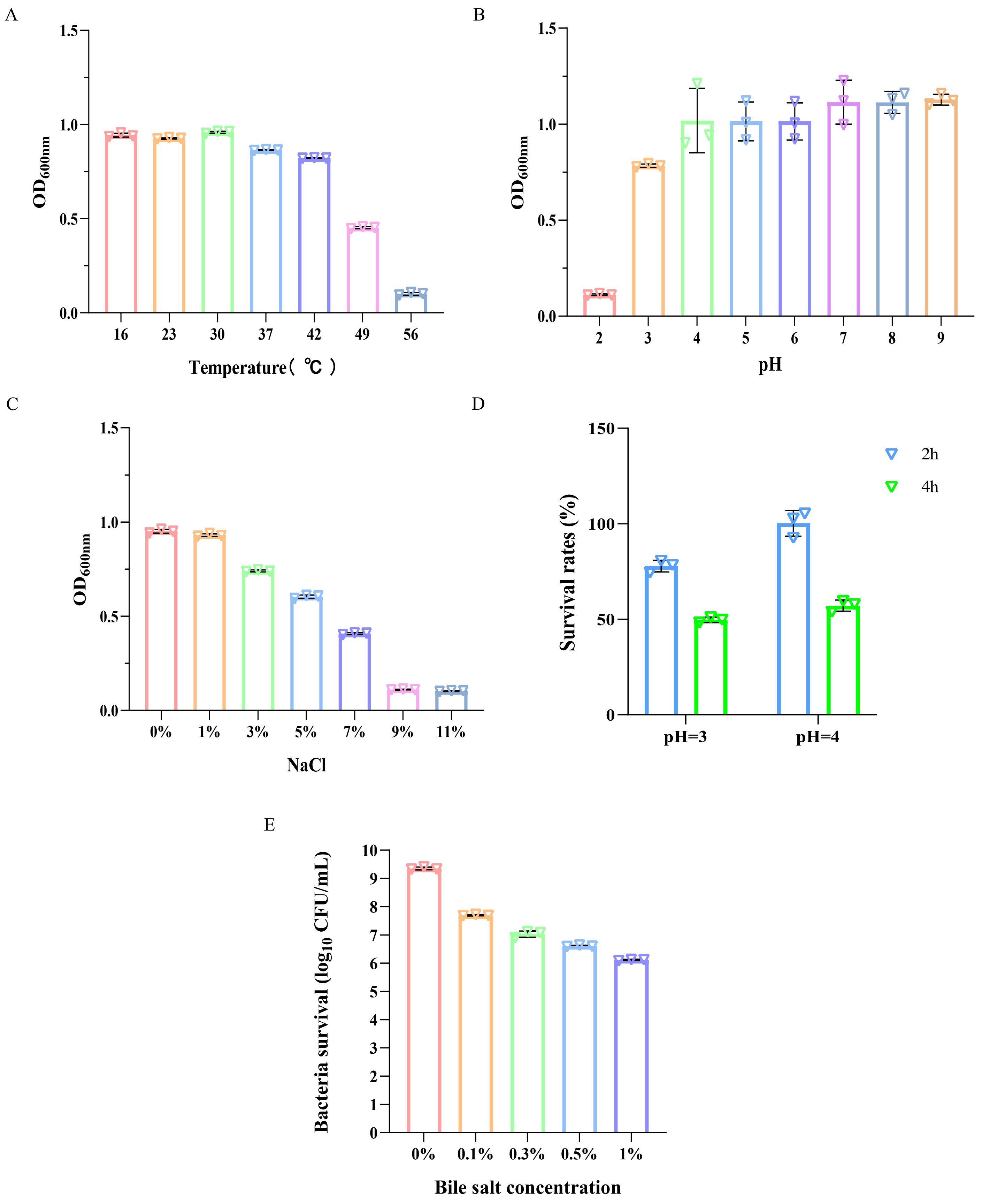

FIG S2 Determination of physiological properties of *L. garvieae* LG1. (A) The growth of *L. garvieae* strain LG1 at different temperatures (16°C, 23°C, 30°C, 37°C, 42°C, 49°C or 56°C). (B) The growth of *L. garvieae* strain LG1 at different pH values (pH=2, 3, 4, 5, 6, 7, 8 or 9). (C) The growth of *L. garvieae* strain LG1 at different NaCl concentrations (0%, 1%, 3%, 5%, 7%, 9% or 11%). (D) The growth of *L. garvieae* strain LG1 in the simulated gastric fluid of different pH values (pH=3 or 4). (E) The growth of strain *L. garvieae* LG1 at different bile salt concentrations (0%, 0.1%, 0.3%, 0.5% or 1%). Graphs are means ±SD from data pooled from three (A-E) biological replicates.
