# Supplementary material for: Gut Lactococcus garvieae promotes protective immunity to foodborne Clostridium perfringens infection
Source: Microbiol Spectr. 2024 Aug 27;12(10):e04025-23. doi: 10.1128/spectrum.04025-23 (PMC11448249; doi:10.1128/spectrum.04025-23)
Supplement: Fig. S3 — Evaluation of the ability of L. garvieae LG1 to adhere to the intestinal mucosal surface. [file spectrum.04025-23-s0003.pdf]

Figure S3

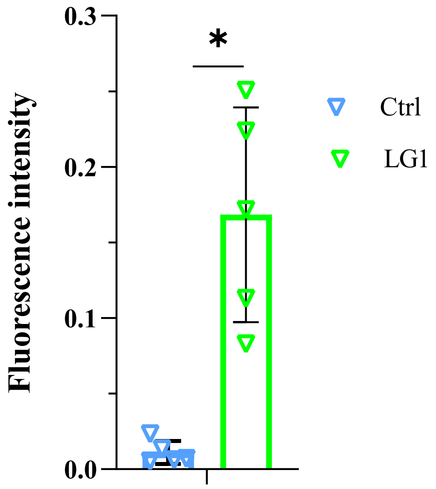

FIG S3. Evaluation of the ability of L. garvieae LG1 to adhere to the intestinal mucosal surface. Six-to eight-week-old wild-type C57BL/6J mice were orally exposed to a mixture of antibiotics (penicillin and streptomycin, 0.5g/kg of body weight, gentamicin, 0.05g/kg of body weight) for 2 days. Two days later, mice were orally administrated with 1 $\times 10^{10}$ CFUs of FITC-tagged *L. garvieae* strain LG1 for 24 h. The mean fluorescence intensity (MFI) of the FITC-positive bacteria were determined. Graphs are means ±SD from data pooled from five biological replicates. Data were considered significant when *p-value < 0.05.
