# Supplementary material for: Gut Lactococcus garvieae promotes protective immunity to foodborne Clostridium perfringens infection
Source: Microbiol Spectr. 2024 Aug 27;12(10):e04025-23. doi: 10.1128/spectrum.04025-23 (PMC11448249; doi:10.1128/spectrum.04025-23)
Supplement: Fig. S4 — The effect of L. garvieae LG1 on the viability of human intestinal epithelial Caco-2 cells. [file spectrum.04025-23-s0004.pdf]

Figure S4

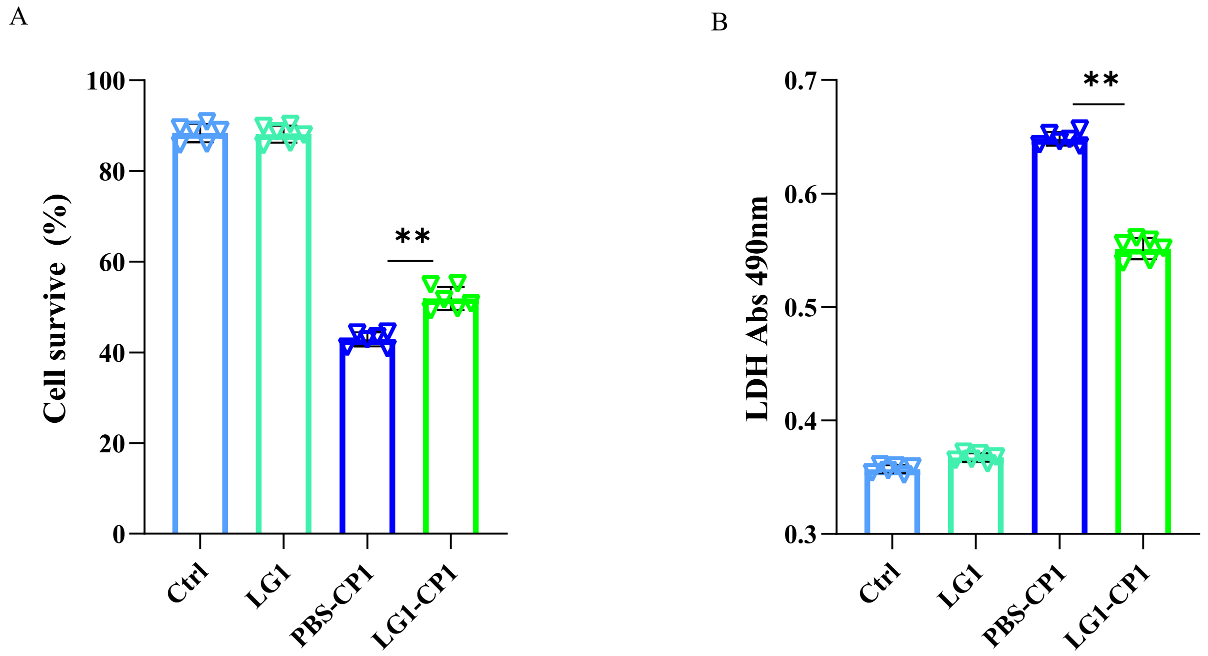

FIG S4. **The effect of *L. garvieae* LG1 on the viability of human intestinal epithelial Caco-2 cells.** The Caco-2 cell were stimulated with *L. garvieae* strain LG1 (MOI=50, 30min) prior to *C. perfringens* strain CP1 (MOI=100, 90 min). (A) Determination of Caco-2 cell viability by Annexin V-FITC/PI staining. (B) LDH release was quantified to monitor cell lysis. Graphs are means ±SD from data pooled from six (A, B) biological replicates. Data were considered significant when ***p*-value < 0.01.
