# Supplementary material for: Gut Lactococcus garvieae promotes protective immunity to foodborne Clostridium perfringens infection
Source: Microbiol Spectr. 2024 Aug 27;12(10):e04025-23. doi: 10.1128/spectrum.04025-23 (PMC11448249; doi:10.1128/spectrum.04025-23)
Supplement: Fig. S5 — The survival curve of the experimental scheme. [file spectrum.04025-23-s0005.pdf]

Figure S5

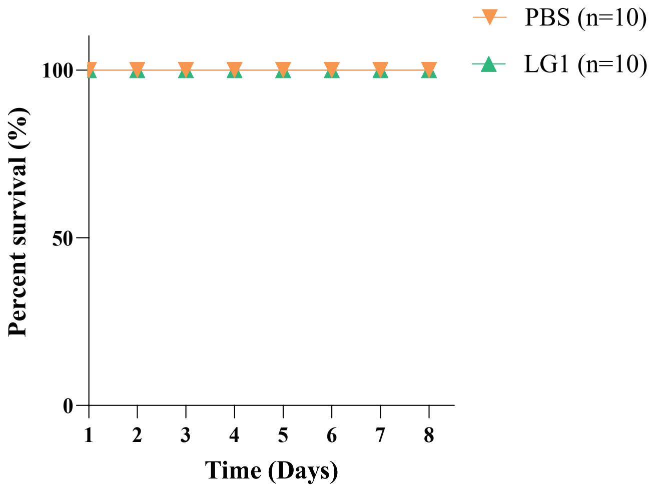

Figure S5. The survival curve of the experimental scheme. Wild-type C57BL/6J mice were fed with PBS or 1×10^{10} CFUs of *L. garvieae* strain LG1 (n=10 for each group).
